# The synergistic effect of titanium dioxide nanoparticles and yeast isolated from fermented foods in reduction of aflatoxin B1

**DOI:** 10.1002/fsn3.3635

**Published:** 2023-08-25

**Authors:** Shohreh Nasiri Poroj, Mohaddeseh Larypoor, Mohammad Reza Fazeli, Farid Shariatmadari

**Affiliations:** ^1^ Department of Microbiology, Faculty of Biological Sciences Islamic Azad University Tehran North Branch Tehran Iran; ^2^ Department of Drug and Food Control, School of Pharmacy Tehran University of Medical Sciences Tehran Iran; ^3^ Department of Poultry Science, Faculty of Agriculture Tarbiat Modares University Tehran Iran

**Keywords:** mycotoxins, probiotics, *Saccharomyces cerevisiae*, TiO_2_

## Abstract

The presence of aflatoxins in food products can lead to health risks in human societies. Therefore, in the present study, the effect of yeast strains isolated from fermented products and titanium dioxide nanoparticles (TiO_2_‐NPs) was studied on aflatoxin reduction. Yeast strains were isolated from fermented products such as sweet fruits and dairy products and identified using biochemical, ascospore (testing by culture medium optimization V8 which is called V8NLF), and molecular methods. The probiotic activity of four selected yeasts was evaluated. Then, the effect of selected yeast isolates and TiO_2_‐NPs on reducing aflatoxin B1 (AFB1) in the medium was studied by measuring AFB1 using ELISA and HPLC. The results of biochemical and molecular identification experiments indicate that the selected strain (Y1) is *Saccharomyces cerevisiae*. The selected strains showed good tolerance to different concentrations of bile salt, pH, and NaCl, indicating appropriate probiotic activity. It also showed antimicrobial activity against *Escherichia coli*, *Shigella dysenteriae*, and *Salmonella typhimurium*. Selected strain and TiO_2_‐NPs showed AFB1 reducing activity in the medium and when combined, showed synergistic effects in reducing AFB1. TiO_2_‐NPs in combination with selected yeast strains have a high ability to remove AFB1 from the medium and, therefore, can be used for future studies.

## Graphical abstract

The presence of aflatoxins in food products can lead to health risks in human societies. Therefore, in the present study, the effect of yeast strains isolated from fermented products and titanium dioxide nanoparticles (TiO_2_‐NPs) was studied on aflatoxin reduction. Yeast strains were isolated from fermented products such as sweet fruits and dairy products and identified using biochemical and molecular methods. Their probiotic potential was measured. Then, the effect of selected yeast isolates and TiO_2_‐NPs on reducing aflatoxin B1 (AFB1) in the medium was studied by measuring AFB1 using ELISA and HPLC. The results showed TiO_2_‐NPs in combination with selected yeast strain have a high ability to remove AFB1 from the medium and, therefore, can be used for future studies.

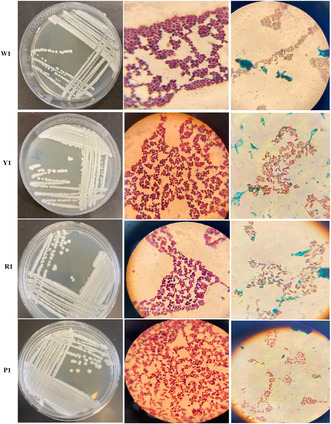

## 1 INTRODUCTION

Aflatoxins are highly toxic and heat‐tolerant secondary metabolites that are difficult to convert to nontoxic substances. Aflatoxins are a series of known sulfur polycyclic compounds that emit fluorescence light when exposed to ultraviolet light (Smith & Groopman, 2018). Among them, aflatoxin B1 (AFB1) is a potent mycotoxin, extremely toxic, mutagenic, and carcinogenic, causing chromosomal defects and hepatoxicity in humans. Together with the hepatitis B virus, it can cause liver cancer in humans (Rawal et al., 2010). An epidemic of diseases caused by the consumption of aflatoxin‐contaminated foods has been observed in some areas of the world, especially in Africa (Daniel et al., 2011). Chemical, physical, and biological methods have been used to reduce aflatoxin (Jalili, 2016; Peles et al., 2021). Physical methods such as heat, ultraviolet radiation, and ionizing radiation have proved to be less effective (Pankaj et al., 2018). Chemical methods include the use of chlorinating, oxidizing, and hydrolytic substances that are not cost‐effective and have adverse health effects (Pankaj et al., 2018). Researchers have found that the best way to detoxify food contaminated with aflatoxins is to use effective microbial species that eliminate the need to use harmful chemicals (Adebo et al., 2017). In this regard, lactic acid bacteria (LAB), probiotic species, fungi, and the yeast *Saccharomyces cerevisiae* have been shown to reduce aflatoxin contamination (Corassin et al., 2013). *Saccharomyces cerevisiae* is a cheap yeast. Studies have reported that this yeast can bind more than 40% of aflatoxins in the environment to their cell wall (Chlebicz & Śliżewska, 2020). Yeasts can bind to various molecules such as toxins and metal ions on the surface of their cell wall due to their binding agent including mannan polysaccharide in the cell wall (Abdolshahi et al., 2019). Studies have shown that the removal of mycotoxins via this method is mostly due to the adhesion of the toxin to the cell wall compounds whereby the dead cells do not lose the ability to bind and can detoxify toxic compounds (Abdolshahi et al., 2018).

The applications of nanomaterials in the medical sciences have been extended in recent years (Sutariya & Pathak, 2014). Most research is currently looking at the effect of nanoparticles on bacteria, fungi, and viruses (Khezerlou et al., 2018). These materials have different chemical and physical properties compared to bulk ones (Anselmo & Mitragotri, 2019). Among metal oxide nanoparticles, titanium dioxide nanoparticles (TiO_2_‐NPs) are the most widely used compared to other nanoparticles in the industry (Ziental et al., 2020). TiO_2_‐NPs are one of the most important and widely used photocatalysts. Due to its strong photocatalytic properties such as high oxidative properties and optical stability along with other advantages such as nontoxicity, cheapness, and availability, it has been proposed as a unique active optical catalyst and widely used in various fields including purification and sterilization of water, removal of air pollution, as well as used in solar cells (Rizwan et al., 2019).

Thus, according to the above‐mentioned points, it seems necessary to study the reduction of mycotoxins by environmentally friendly methods in today's world. In this study, we tried to investigate the synergistic effect of the yeasts isolated from fermented foods and their combined use with TiO_2_ nanoparticles to reduce AFB1.

## 2 MATERIALS AND METHODS

### 2.1 Yeast isolation and identification

Fermented foods such as whey, pickles, yogurt, raisins, and sweet fruits including apples, white and red grapes, persimmons, plums, and peaches were used to separate the yeast. To this end, 1 g of each sample was dissolved in 90 mL of normal saline and then serially diluted in peptone water (0.1%) to 10^−6^. The prepared dilutions were placed in a culture medium containing Sabouraud Dextrose Broth (SDB) agar. Isolation of yeast strains was performed in yeast extract glucose chloramphenicol (YGC) medium where the pure culture of isolated yeasts was transferred to rich YGC agar medium for 30 days. For long‐term storage of the isolates, the aqueous solution of 10% glycerol (Crespo et al., 2000; Uzunova‐Doneva & Donev, 2002) was used at −70°C, while SDB agar was used for storage at 4°C (Bueno & Gallardo, 1998).

The yeasts were identified by a microscope, plus morphological and biochemical tests including growth in malt extract, high temperature (37°C), as well as the competence to break down fermented sugars (lactose, maltose, sucrose, and glucose).

A handmade V8 medium was applied to observe ascospores in the studied samples. In general, V8 medium includes fresh vegetables. To optimize this culture medium, several different protocols were used, which were as follows.

1. Variety in the type of vegetables: Initially, two different groups of vegetables were used to optimize the culture medium. The vegetables of the first group included 350 g of tomatoes, 350 g of carrots, 150 g of celery, 200 g of beets, 150 g of bell peppers, 150 g of cabbage, 150 g of turnips, 100 g of onions, 50 g of garlic, and 50 g of broccoli. The second group included 400 g of carrots, 200 g of celery, 400 g of tomatoes, 250 g of beetroot, 200 g of peppers, 200 g of spinach, 200 g of lettuce, and 100 g of parsley. Finally, to make 1000 mL of V8 culture medium, 250 g of total vegetables with 3 g of calcium carbonate (to prevent acidification of the medium and hydrolysis of agar) plus 15 g of agar with pH 7 were used.
2. Variety in sterilization method: To optimize the culture medium, the above compounds were once sterilized in an autoclave at 121°C for 20 min. Once again, each vegetable was passed through an MF‐Millipore™ Membrane Filter (0.22 μm, Merck, Germany) separately whereby no heat was used for sterilization.
3. Use of pulp and pulp‐free vegetables: To observe the effect of vegetable pulp, each of the above media was synthesized in two ways: pulp and pulp‐free (vegetable juice).

To observe ascospores from fresh yeast colonies on cultured media, cultures were isolated and the isolates were incubated at 30°C for 5–7 days. Finally, after preparing the expansion from the colonies and fixing it with heat, it was stained with green malachite (10 min with heat) and safranin.

### 2.2 Biochemical tests

For the catalase test, a drop of 3% hydrogen peroxide was poured on a clean slide, and then with a sterile applicator, a quantity of fresh yeast colony (24‐h culture) was dissolved in it. This experiment was repeated with 10% hydrogen peroxide. In the end, the production or nonproduction of oxygen gas bubbles was investigated.

In order to check the resistance of the studied yeasts to cycloheximide, a fresh yeast colony (cultured for 48 h) was cultured in MRS broth medium containing 1% cycloheximide and heated for 48 h at 30°C. Finally, the growth of yeasts in this environment was investigated.

In the sugar fermentation test, phenol‐red culture medium with 1% of different carbohydrates including glucose, lactose, galactose, fructose, trehalose, dextrin, sucrose, maltose, mannitol, mannose, xylose, arabinose, amylose, cellobiose, inulin, maltase, raffinose, ribose, salicin, and sorbitol was prepared and cultured from a fresh yeast colony (48‐h culture) in the medium and heated at 30°C for 24 h. In the preparation of this culture medium, due to the fact that carbohydrates cannot be autoclaved, they were filtered separately with a Millipore 0.45‐μm filter and added to the culture medium. After 24 h, the ability to ferment different carbohydrates was confirmed by checking the color change of the culture medium.

### 2.3 Evaluation of probiotic activities of isolated yeasts

The probiotic activity of the isolated yeasts was evaluated using viability test at different pHs, bile, and NaCl tests.

For viability test at different pHs (1, 2, 3, 4, 5, 6, 7, 8, and 9), SDB with different pHs (pH adjusted by HCl and 5% NaOH) was prepared and then 100‐μL isolated yeast suspensions were cultured at 30°C for 48 h. Finally, the turbidity of the culture medium was measured by a spectrophotometer at 600‐nm light absorption and finally, the growth chart was drawn (Chen et al., 2015).

For the bile tolerance test, SDB was prepared with different concentrations of ox gall (0.1%–0.3%–0.6%–0.9%–1.2%) on pH 6.5. Next, 100 μL of the isolated yeast suspensions was cultured in these media and incubated at 37°C for 48 and 72 days. Finally, the turbidity of the culture medium was measured by a spectrophotometer (Moradi et al., 2018).

Survival of isolated yeast was examined through the incubation of the strain (cultured in the SDB) at different temperatures (20, 25, 30, 37, and 40°C) for 72 h.

### 2.4 Antibiotic susceptibility tests

Yeast cells were cultured at 10^7^ CFU/mL on YGC agar. Standard antibiotic disks (Mast) containing gentamicin (10 μg), vancomycin (10 μg), amoxicillin (10 μg), ciprofloxacin (10 μg), and ampicillin (10 μg) were placed on the YGC agar surface. The plates were incubated for 24 h at 30°C and then, the inhibition zone was measured.

### 2.5 Antimicrobial activity of yeast against bacterial pathogens

Antimicrobial activities of the isolated yeast on pathogenic bacteria were measured by culturing the isolated yeast in an SDB medium and incubating it for 48 h. It was then centrifuged for 15 min at 1,800*g* and the supernatant was filtered. Next, the antimicrobial activities of the metabolites produced by the yeast were measured by the Wells method against including *Salmonella enterica* (PTCC: 1230), *Escherichia coli* (ATCC: 25992), and *Proteus vulgaris* (ATCC 43071). For this purpose, the wells with a volume of 50 μL (2 mm diameter) were drilled on Moller agar medium. Then, 100 μL of isolated yeast suspensions (filtered by Millipore filter) were added to each well and after 24 h, the diameter of the growth inhibition zone was accurately measured using a ruler. We used doxycycline as a control. All the experiments were repeated three times (*n* = 3).

### 2.6 NaCl tolerance test

SDB supplemented with 4.5%–6.5% NaCl was prepared where the positive samples in the above experiment were incubated at 30°C for 48 h. Finally, the turbidity of the culture medium was measured by a spectrophotometer (*n* = 3).

### 2.7 Proteolytic activities of isolated yeasts

The proteolytic activity of separated yeasts was performed by culturing 20 μL of yeast suspensions on skim milk agar medium and incubating it at 30°C for 3 days (*n* = 3). The colonies that zone around them were considered as strains with proteolytic activity.

### 2.8 Molecular identification by polymerase chain reaction (PCR) and sequencing

Molecular identification was performed using the internal transcribed spacer region (ITS, 5′‐TCCGTAGGTGAACCTGCGGCGG‐3′) and large subunit (LSU) of the yeast nuclear ribosomal RNA (rRNA, 5′‐GGTCCGTGTTTCAAGACGG‐3′) gene complex. First, DNA was extracted by DNA extraction kit (Pishgaman Enteghal Gene) according to the manufacturer's instructions. Master Mix included 0.2‐μL Tag DNA Polymerase, 3‐μL Tris–HCL, (NH4)_2_SO_4_, 1.5‐μL MgCl_2,_ and 0.4‐μL dNTP. The reaction mixture consisted of 12.5‐μL Master Mix, 0.5 μL each of forward and reverse primers, 1‐μL DNA, and 10.5‐μL distilled water in a final volume of 25 μL. The temperature–time program of the device includes 1 cycle of 95°C for 5 min, 35 cycles of 95°C for 30 s, 58°C for 45 s, and 72°C for 60 s, and a final cycle of 72°C for 5 min. Finally, PCR products were sent to Iran Biological Resources Center (Tehran, Iran) for sequencing and the results were blasted with the sequences in the GeneBank database.

### 2.9 In vitro study of the effect of yeast and TiO_2_‐NPs on AFB1

#### 2.9.1 Preparation of the standard AFB1

AFB1 purchased from Sigma (Germany) was dissolved in a benzene–acetonitrile organic solvent (98:2 v/v) to reach 25 μg/mL concentrations according to the manufacturer's instructions. Phosphate buffer (1:10 [v/v], pH 7.4) was used to dilute the sample. To remove the organic solvent, a water bath was used at 80°C for 15 min.

#### 2.9.2 Preparation of aflatoxin from *Aspergillus flavus*

Initially, *A. flavus* (PTCC 5018) purchased from Iran Scientific and Industrial Research Center was cultured in PDB medium in several flasks and incubated at 26°C for 2 weeks. To extract aflatoxin from PDB medium, the contents of each flask were first mixed homogeneously. The contents of the flasks were then passed through a Whatman 42 paper filter (with a porosity of 2–3 μm). For every 100 mL of filtered solution, 40 mL of chloroform solvent was added and the resulting mixture was stirred in a decanter funnel for 20 min. After 24 h, the lower phase containing chloroform solvent and aflatoxin was isolated. The solvent was separated by a rotary apparatus at 45°C under a vacuum. The residue was dissolved in 10 mL of HPLC purity methanol solvent (Shimadzu, Japan) and passed through a 0.22‐μm nozzle filter (Sigma). The concentrated sample was stored in a freezer at −20°C. Then, HPLC was performed for qualitative identification and quantitative measurement of AFB1 (Martins et al., 2003). The device was equipped with a fluorescent detector and an ODS Spheri‐5 Brownlee column, and the mobile phase (water–acetonitrile–methanol) was set at a flow rate of 1 mL/min.

#### 2.9.3 Measurement of free and bounded to *S. cerevisiae* AFB1

The yeast was cultured in SDB broth medium, and after 48 h (growth turbidity was measured via spectrophotometry at 600 nm), the tubes containing the yeast were centrifuged for 15 min at 1800*g*. The yeast precipitate was washed three times with 5 mL of phosphate buffer solution (PBS) and then, 2‐mL standard and extracted AFB1 solution (50 μg) were added in separate vials. AFB1 samples in PBS were used as a control. Falcons were incubated for 48 h at 30°C. The samples were collected at different time intervals (0, 24, and 48 h) after which each sample was centrifuged for 15 min at 1800*g* to measure free aflatoxin. The samples were screened with an AFB1 ELISA kit (ZellBio) where the optimal sample was analyzed by HPLC. Finally, the percentage of AFB1 bound to yeast was calculated.

#### 2.9.4 Measurement of the binding of TiO_2_ nanoparticles to aflatoxin B1

TiO_2_‐NPs used in the current study were purchased from Nanopooyeshyekta Corporation (Tehran, Iran). The features of TiO_2_‐NPs included an average geometric diameter of 10–25 nm, a purity of over 90%, and an anatase type.

Different concentrations of TiO_2_‐NPS (0.05, 0.1, and 0.15 mg/mL) were prepared and subjected to ultraviolet radiation for 120 min. Then, 4 mL of standard and extracted AFB1 solution (100 μg) was added and incubated in separate vials for 48 h at 30°C. The samples were collected at different time intervals (0, 24, and 48 h) and then each was centrifuged for 15 min at 1800*g* to measure free aflatoxin and the supernatant was screened with an AFB1 ELISA kit, and the optimal sample was analyzed by HPLC. Finally, the percentage of aflatoxin bound to TiO_2_‐NPs was calculated.

To investigate the effect of pH, the optimal concentration of TiO_2_ NPs (obtained in the previous step) was added to 5 mL of standard and extracted AFB1 solution at pH 5 and 8 for 24 h. The samples were centrifuged for 15 min at 1800*g* to measure free aflatoxin and the supernatants were screened with an AFB1 ELISA kit and the optimal sample was analyzed by HPLC.

#### 2.9.5 Measurement of the synergic effect of isolated yeast and TiO_2_‐NPs in adsorption of AFB1 toxin

*Saccharomyces cerevisiae* (Exir 98) was cultured in SDB medium for 48 h. After centrifugation for 15 min at 1800*g*, the yeast supernatant was added to 5 mL of AFB1 solution (benzene–acetonitrile organic solvent: AFB1 98:2 v/v, 125 μg). Also, TiO_2_‐NPs (with the optimal concentration obtained in the previous experiment) were added to the solution and incubated for 24 h at 30°C. After centrifugation for 15 min at 1800*g*, the supernatant was analyzed by HPLC to measure free aflatoxin, and finally, the amount of aflatoxin bound to the nanoparticle yeast was calculated.

### 2.10 Statistical analysis

Data were expressed as means ± SD. A two‐way analysis of variance was used for statistical analysis. SPSS software version 26 was used for data analysis. Tukey's multiple range test was applied to compare the means. *p* < .05 was considered significant.

## 3 RESULTS

### 3.1 The isolation and identification of yeast

**FIGURE 1:**
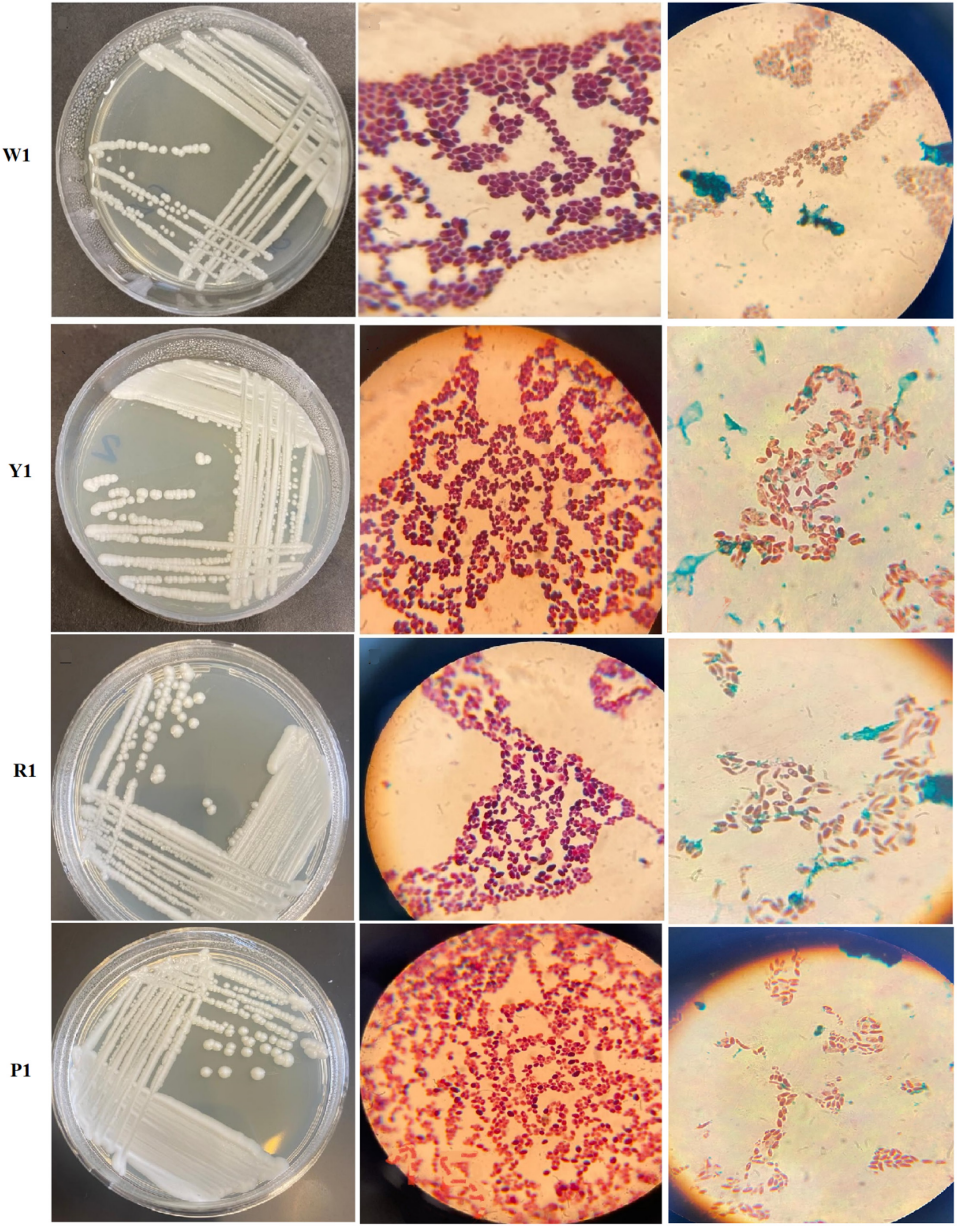
The photographs of the four isolated *Saccharomyces* spp. colonies morphology on yeast extract glucose chloramphenicol agar medium. (Left), microscopic image (1000 ×) of Gram‐stained smears illustrating the Gram‐positive. (Middle) and microscopic image (1000 ×) of ascospore (Right).

A total of 41 strains out of 50 samples were identified using microbiological and biochemical tests, and four strains (isolated from whey [W1], yogurt [Y1], raisins [R1], and peaches [P1]) were identified as *Saccharomyces* genus. These four strains were selected for the next stages of the experiment. Two different vegetable combinations were applied to optimize the culture medium. The composition of the first group of vegetables containing tomatoes, carrots, celery, beets, bell peppers, cabbage, parsley, onion, and garlic had better results than the composition of the second group of vegetables. The results revealed that ascospore production in vegetables of the first group was greater and faster. The method of sterilization of the culture medium had a great effect on its performance whereby no colony was observed in the vessel medium that used Millipore filter to sterilize it. Thus, the best way to sterilize the culture medium is to use a 121°C autoclave for 20 min. The vessel environment, which was made from vegetable juice (without pulp), was both structurally better and produced more ascospores. As a result, the highest growth, sexual reproduction, and ascospore production were observed in the environment made of autoclaved vegetables without pulp, including tomatoes, carrots, celery, beets, bell peppers, cabbage, parsley, onions, and garlic. Optimization of this culture medium with this method and compounds has been done for the first time and is introduced as V8NLF culture medium. Figure 1 depicts the isolated strains. The selected strain could grow in V8NLF medium.

### 3.2 Biochemical tests

Catalase and 0.1% and 0.01% cycloheximide tests were performed on selected yeast strains. The results indicated that the fermentation of sugars including maltose, raffinose, galactose, sucrose, glucose, sorbitol, trehalose, and inositol was done by the yeast isolate. However, no fermentation occurred in the presence of the sugars such as arabinose, lactose, mannitol, xylose, cellobiose, rhamnose, dextrin, and salicin. Absorption of carbon compounds such as raffinose, galactose, sucrose, glucose, arabinose, sorbitol, and inositol was also observed in the selected isolates. However, no uptake of potassium nitrate and sodium nitrate was found. The isolated yeast strains were positive for catalase but negative for urea and cycloheximide 0.1% and 1%, respectively.

### 3.3 Evaluation of probiotic activities

**FIGURE 2:**
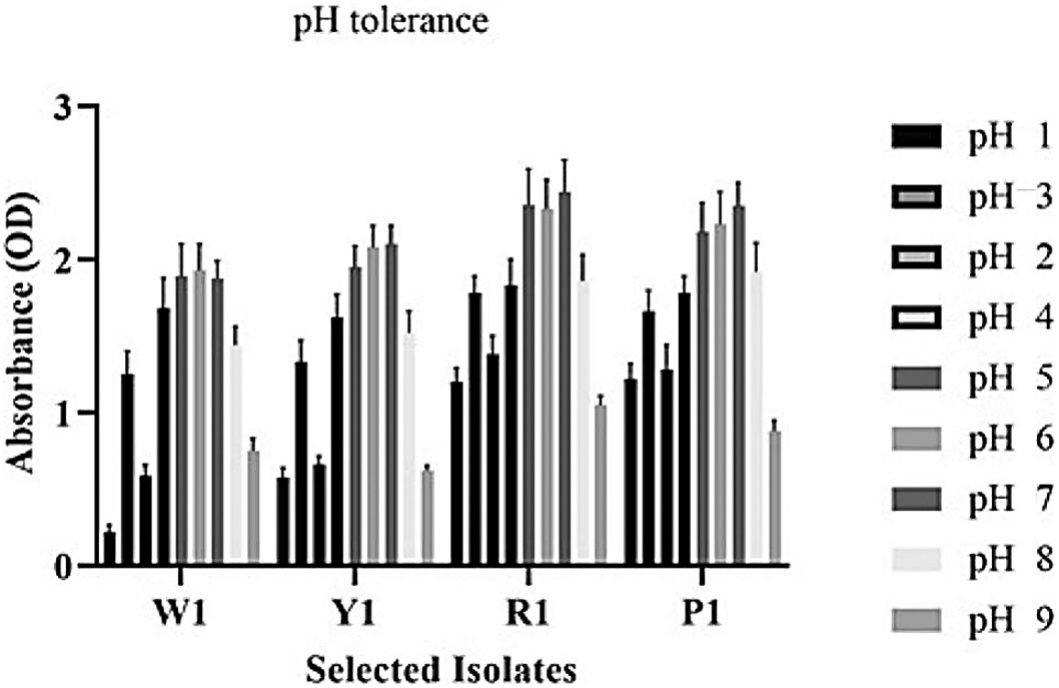
The growth of isolated yeast strains (W1, Y1, R1, and P1) at different medium pHs ranging from 1 to 9. The chart shows high tolerance of the R1 and P1 yeast strains to a wide range of pH from 1 to 9, suggesting the high resistance of yeast isolate to acidic and alkaline conditions of the gastrointestinal tract. Nevertheless, the highest yeast growth was obtained at pH 5, 6, and 7, while the lowest growth was seen at pH 1 and 9.

The results of the present study indicated high tolerance of the R1 and P1 yeast strains to a wide range of pH from 1 to 9, suggesting the high resistance of yeast isolate to acidic and alkaline conditions of the gastrointestinal tract. Meanwhile, the highest yeast growth was obtained at pH 5, 6, and 7, while the lowest growth was seen at pH 1 and 9 (Figure 2).

**FIGURE 3:**
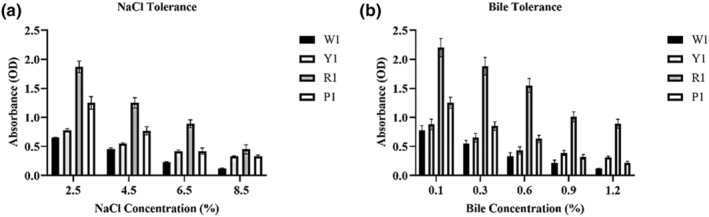
The growth of yeast isolates at different concentrations of NaCl (a) and ox gall (b). Although the growth of yeast isolates diminished in the medium with increasing NaCl percentage, the results showed good tolerance of R1 isolate to high salt concentrations (a). Also, high tolerance of R1 yeast strain to bile salt was observed at different concentrations of ox gall (b).

Although the growth of yeast isolates diminished in the medium with increasing NaCl percentage, the results showed good tolerance of R1 isolate to high salt concentrations (Figure 3a). Also, high tolerance of R1 yeast strain to bile salt was observed at different concentrations of ox gall (Figure 3b).

The isolates could grow at temperatures of 25, 30, 37, 40, and 45°C, but the growth diminished at temperatures of 40 and 45°C.

### 3.4 Molecular identification

**FIGURE 4:**
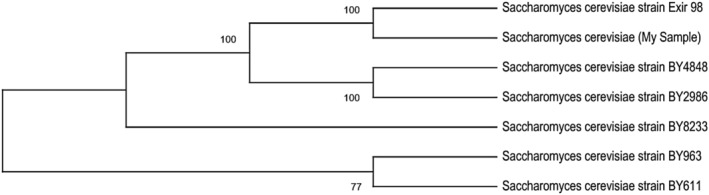
The results of blasting of sequences of the internal transcribed spacer region and large subunit of the yeast nuclear ribosomal RNA gene complex with 6 strains. The BLAST results revealed that the Y1 belongs to *Saccharomyces cerevisiae* strain Exir 98, which showed 100% identity.

The sequencing of the ITS region and LSU of the yeast nuclear rRNA gene complex was done to identify the Y1 yeast isolates. The results showed that the Y1 belongs to *S. cerevisiae* strain Exir 98, which showed 100% identity (Figure 4). The cluster analysis showed the lowest identity of the Y1 selected isolate with BY611 and BY963 strains (77%), indicating the largest differences.

### 3.5 Antimicrobial activities of the selected yeast isolate

The results of antimicrobial activity using the disk diffusion method revealed that the Y1 isolate showed an inhibitory effect against *E. coli* (12.35 ± 2.22 mm), *Shigella dysenteriae* (10.38 ± 1.75 mm), and *Salmonella typhimurium* (11.68 ± 3.12).

In the antibiotic susceptibility test, the tested isolate was resistant to gentamicin, vancomycin, amoxicillin, ciprofloxacin, and ampicillin antibiotics.

### 3.6 Removal of AFB1 by the yeast isolate and TiO_2_‐NPs

**FIGURE 5:**
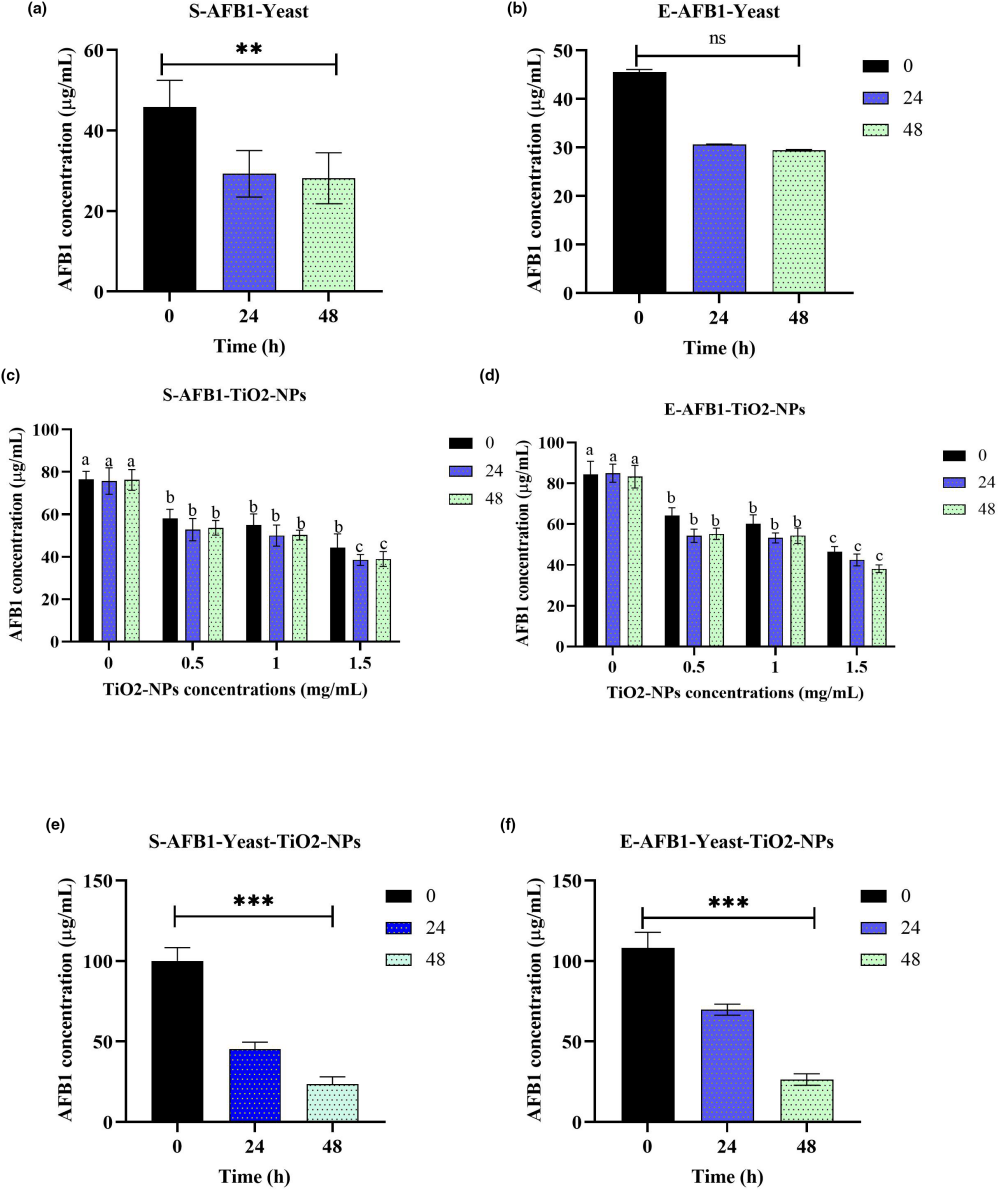
The effects of yeast (a) and (b) and TiO2‐NPs (c) and (d) on the standard and extracted AFB1. (e) and (f) the synergistic effects of yeast and TiO2‐NPs on standard and extracted AFB1, respectively. The results revealed that the isolated yeast strain after 24 h could absorb and reduce standard AFB1 up to 38.80% and AFB1 extracted from *Aspergillus flavus* up to 38.04% (a) and (b). TiO2‐NPs at different concentrations reduced the contents of standard and extracted. It seems that the concentration of 1.5 μg/mL had the greatest effect on reducing the AFB1 content by reducing 49.34% of the standard aflatoxin and 45.23% of the extracted aflatoxin (c) and (d). When yeast isolate was used in combination with TiO2‐NPs, it showed synergistic effects in reducing standard and extracted AFB1 (e) and (f). ** Increasing absorption of aflatoxin‐B1 by yeast based on time, *** The maximum amount of absorption of aflatoxin‐B1 by the whole nanoparticle based on time.

The results of the present study indicated that the isolated yeast strain was able to reduce standard AFB1 by approximately 50% and AFB1 extracted from *A. flavus* after 24 h (Figure 5a,b). The optimal sample (24 h) was examined by HPLC. The results showed that the desired yeast can adsorb and reduce standard AFB1 up to 38.80% and extracted AFB1 up to 38.04%.

TiO2‐NPs at different concentrations reduced the contents of the standard and extracted. It seems that the concentration of 1.5 μg/mL had the greatest effect on reducing the AFB1 content by lowering 49.34% of the standard aflatoxin and 45.23% of the extracted aflatoxin (Figure 5c,d).

When yeast isolate was used in combination with TiO_2_‐NPs, it showed synergistic effects in reducing the standard and extracted AFB1 (Figure 5e,f).

## 4 DISCUSSION

The probiotic activity of yeasts has been shown in various studies (Cho et al., 2018; Gut et al., 2019), noting the importance of the discovery of new strains of yeast with probiotic activity. Thus, in the present study, we tried to isolate new strains from fermented food products including whey, pickles, yogurt, raisins, and sweet fruits including apples, white and red grapes, persimmons, plums, and peaches, where the selected strain was studied in terms of probiotic activities. There are several diagnostic tests to isolate *S. cerevisiae* from other yeasts (Vilela et al., 2020). One of the most important characteristics of *Saccharomyces* is the ability to produce ascospores from sexual reproduction. Ascospores are not produced in conventional fungal culture media (Iacob et al., 2016). To produce yeast ascospores, they must be grown in a special culture medium containing vegetables. Since a single formulation was not available for this culture medium, we optimized the V8NLF medium for the first time in this study. This new formulation would facilitate the detection of ascospores and would be both time and cost‐saving. The results showed that the isolated strain of yeast had good probiotic activity. Due to the high sensitivity of molecular methods to identify the desired strain (Arbefeville et al., 2017), we used the sequencing of ITS region ITS and LSU of the yeast nuclear rRNA gene complex, with the results showing that the desired strain was *S. cerevisiae* strain Exir 98.

One of the important criteria for the probiotic activity of microorganisms is their ability to adapt to gastrointestinal conditions (Menezes et al., 2020). Thus, in the present study, the selected yeast isolates were exposed to different concentrations of bile salt, as well as acidic and base pHs as well as NaCl concentrations. The results of the present study showed the proper growth of the yeast isolate at high concentrations of bile (1.2%), tolerance to acidic (1.5) and basic (9) pHs, and the selected strain also showed proteolytic activity, suggesting the probiotic activity of the selected yeast strain, which can be used as a probiotic strain in the food industry. The survival of other strains of yeast has been reported in other studies (de Oliveira et al., 2019; Reyes‐Becerril et al., 2021), which is similar to the current findings.

In the present study, the antimicrobial activity of the selected yeast strains was studied against *Salmonella enterica* (PTCC: 1230), *E. coli* (ATCC: 25992), and *Proteus vulgaris* (ATCC 43071). The results indicated that the selected strain had high antimicrobial activity against three gastrointestinal pathogens. The yeast antimicrobial activity has been reported in other studies (AbdElatif et al., 2016; Younis et al., 2017). The antimicrobial activity of yeasts can be attributed to the production of compounds such as volatile thermolabile toxic extract (Viljoen, 2006), antilisterial hydrophobic peptides, and mycocins (Chen et al., 2015; Hatoum et al., 2013). On the other hand, the expression of the khs killer gene reported in *S. cerevisiae* may be the antimicrobial mechanism of yeast against bacteria (de Ullivarri et al., 2014).

AFB1 is one of the mycotoxins whose toxic effects are well known (Li et al., 2018). In recent years, with the increasing knowledge of mycotoxins and their adverse effects on humans and animals, many efforts have been made to remove these toxins from food (Egmond et al., 2007). Today, the use of toxin binders to remove fungal toxins from food has been widely researched whose most common use is in livestock and poultry feed to bind to mycotoxins and prevent their absorption in the gastrointestinal tract (Atiqul et al., 2020). In the present study, it was shown that the selected yeast strain could reduce standard and extracted AFB1 in vitro. Over 95% reductions in aflatoxin in the medium by other yeasts such as *Candida krusei* and *Pichia anomala* have been reported (Deepak, 2015; Niknejad et al., 2012). Yeasts are important because of their advantages such as simple nutritional requirements, ability to grow in fermenters in the presence of inexpensive culture medium, ability to survive in different environmental conditions, and nonproduction of toxic compounds. They are also used as biological tools to control microbial contaminants and their toxic metabolites in food (Hassan et al., 2021). It has been reported that the cell wall of yeasts contains polysaccharides such as glucan and mannan, which bind to AFB1 by hydrogen, ionic, and hydrophobic bonds and reduce the concentration of AFB1 in the medium (Madrigal‐Bujaidar et al., 2015). Thus, the reduction of standard and extracted AFB1 observed in the present study can be attributed to the binding of AFB1 to the yeast cell walls. *Saccharomyces cerevisiae* has been shown to reduce the severity of aflatoxicosis by chelating, binding to aflatoxin molecules, and removing it from the gastrointestinal tract (Zhao et al., 2010). It has also been found that the addition of mannooligosaccharide extracted from the cell wall of *S. cerevisiae* could reduce aflatoxin by 79% (Sun et al., 2015). Based on sampling at different times in the present study, it can be concluded that the binding of aflatoxin to yeast is rapid.

In recent years, the use of nanoparticles has increased in many sectors including medicine, industry, agriculture, water purification, packaging, cosmetics, etc. (Gade et al., 2010). Among them, TiO_2_‐NPs have received massive attention due to their unique properties and nontoxicity. In the present study, it was shown that TiO_2_‐NPs were able to reduce standard and extracted AFB1 at 1.5 mg/L. It seems that the TiO_2_‐Nps structure can adsorb AFB1 to its surface and reduce its concentration in the medium. The antifungal and aflatoxin‐reducing effects of TiO_2_ nanoparticles have been reported in other studies (Davarani et al., 2018; Sun et al., 2021), which is in line with the results of the present study.

The synergistic effect of selected yeast strain along with TiO_2_‐NPs was shown in reducing standard and extracted AFB1 in the medium. To the best of our knowledge, this is the first study to examine the effect of a combination of yeast strain and TiO_2_‐NPs in reducing AFB1. It seems that yeast cell wall monosaccharides and the unique properties of TiO_2_‐NPs led to a sharp decline in AFB1 in the culture medium.

In general, it can be concluded that the selected yeast strain had probiotic properties and could reduce AFB1 in the medium, and when used in combination with TiO_2_‐NPs, it showed a synergic effect on reducing AFB1 in the medium. Nevertheless, in vivo studies are required to confirm its effectiveness.

## AUTHOR CONTRIBUTIONS

**Shohreh Nasiri Poroj:** Data curation (equal); formal analysis (equal); funding acquisition (lead); investigation (lead); methodology (equal); resources (lead); software (lead); validation (equal); visualization (equal); writing – original draft (lead); writing – review and editing (equal). **Mohaddeseh Larypoor:** Conceptualization (equal); data curation (equal); formal analysis (equal); project administration (lead); writing – review and editing (equal). **Mohammad Reza Fazeli:** Conceptualization (equal); formal analysis (equal); data curation (equal); writing – review and editing (equal). **Farid Shariatmadari:** Project administration (equal); writing – review and editing (equal).

## FUNDING INFORMATION

The authors declare no specific funding for this work.

## CONFLICT OF INTEREST STATEMENT

The authors have declared no conflict of interest.

## ETHICS STATEMENT

Not applicable.

## Data Availability

All data generated or analyzed during this study are included in this published article. Raw sequence data on 16 s RNA gene had been submitted to the NCBI Sequence Read Archive (SRA) with the accession number PRJNA733868 (https://www.ncbi.nlm.nih.gov/sra/PRJNA733868).
